# Multicentric solid pseudopapillary neoplasms of the pancreas diagnosed by endoscopic ultrasound-guided fine needle aspiration: a case report

**DOI:** 10.1186/s40792-015-0111-8

**Published:** 2015-10-27

**Authors:** Megumi Yamaguchi, Toshikatsu Fukuda, Masahiro Nakahara, Mio Amano, Daisuke Takei, Masumi Kawashima, Yusuke Sumi, Hironobu Amano, Shuji Yonehara, Keiji Hanada, Toshio Noriyuki

**Affiliations:** Department of Surgery, Onomichi General Hospital, 1-10-23, Hirahara, Onomichi, Hiroshima 722-8508 Japan; Department of Gastroenterology, Onomichi General Hospital, 1-10-23, Hirahara, Onomichi, Hiroshima 722-8508 Japan; Department of Diagnostic Pathology, Onomichi General Hospital, 1-10-23, Hirahara, Onomichi, Hiroshima 722-8508 Japan; Faculty of Medicine, Hiroshima University, 1-2-3, Kasumi, Hiroshima 734-8551 Japan

**Keywords:** Multicentric, Solid pseudopapillary neoplasm (SPN), Endoscopic ultrasound-guided fine needle aspiration (EUS-FNA)

## Abstract

Solid pseudopapillary neoplasm (SPN) of the pancreas is a rare tumor. This neoplasm usually arises as a single mass; multicentricity is exceptionally rare. We report the preoperative diagnosis of multicentric SPNs by endoscopic ultrasound-guided fine needle aspiration (EUS-FNA). A 32-year-old woman presented to the hospital with a pancreatic tumor that was detected on abdominal echography. Contrast-enhanced computed tomography (CT) scans revealed a 5-mm low-density mass in the body of the pancreas and a 10-mm mass in the tail of the pancreas. Magnetic resonance imaging (MRI) also revealed two tumors in the body and tail of the pancreas. On endoscopic ultrasonography (EUS), two indistinct and heterogeneous echogenic masses were found, and EUS-FNA was performed for each of these tumors. Cytological analysis revealed that the two masses were highly cellular with papillary groups of small, uniform, oval cells surrounding a fibrovascular core. Immunohistochemistry was positive for α-1 antitrypsin, vimentin, neuron-specific enolase (NSE), CD10, and progesterone receptor. These features confirmed the preoperative diagnosis of multicentric SPNs. The patient underwent laparoscopic distal pancreatectomy with splenectomy. The final pathologic diagnosis was multicentric SPNs. During 2 years of follow-up, she has not developed any recurrence.

## Background

Solid pseudopapillary neoplasm (SPN) of the pancreas is rare, accounting for approximately 0.13–2.7 % of all pancreatic tumors and 1–2 % of all exocrine pancreatic tumors [1]. SPN usually occurs in young women and has low potential for malignancy. In rare cases, it may spread to the lymph nodes, resulting in distant metastasis. Therefore, complete surgical resection is the main treatment for SPN.

SPN is usually a solitary mass, and multicentricity is exceptionally rare. This case report describes the preoperative diagnosis of multicentric SPNs by endoscopic ultrasound-guided fine needle aspiration (EUS-FNA).

## Case presentation

A 32-year-old woman presented to our hospital with a pancreatic body tumor, which was identified by abdominal echography during health screening. No symptoms were evident, and her medical history was unremarkable.

**Fig. 1:**
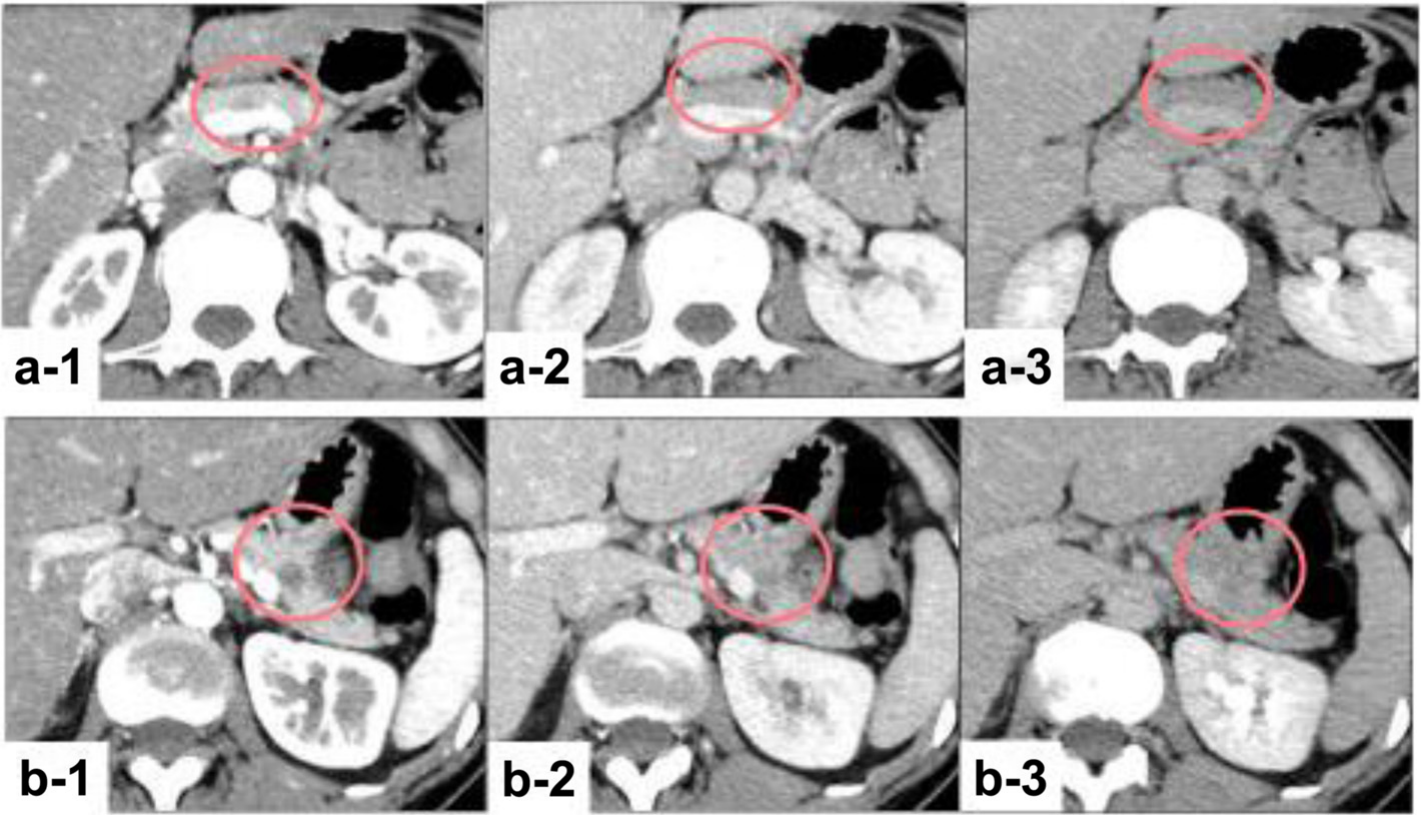
Contrast-enhanced computed tomography scan. (*a-1*) There was a 5-mm low-density mass in the pancreatic body. (*a-2*, *3*) This mass was gradually enhanced in the portal vein phase and the late phase. (*b-1*) There was also a 10-mm low-density mass in the pancreatic tail. (*b-2*, *3*) This mass had the same density and enhancement as the other mass

A contrast-enhanced computed tomography (CT) scan revealed a 5-mm low-density mass in the pancreatic body, which was gradually enhanced in the portal vein phase, and a 10-mm mass in the pancreatic tail, which had the same density and enhancement as the other mass (Fig. 1).

On magnetic resonance imaging (MRI), the two tumors in the body and tail of the pancreas had lower signal intensity on T1-weighted images and higher signal intensity on T2-weighted and diffusion-weighted images. There was no stenosis of the main pancreatic duct (MPD). We observed neither dilatation of the distal side of the pancreatic duct nor communication between the tumors and the MPD.

**Fig. 2:**
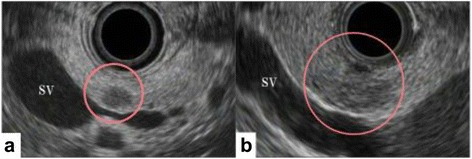
Endoscopic ultrasonography revealed indistinct and heterogeneous echogenic masses in the pancreatic body and tail. **a** body, **b** tail. *SV* splenic vein

**Fig. 3:**
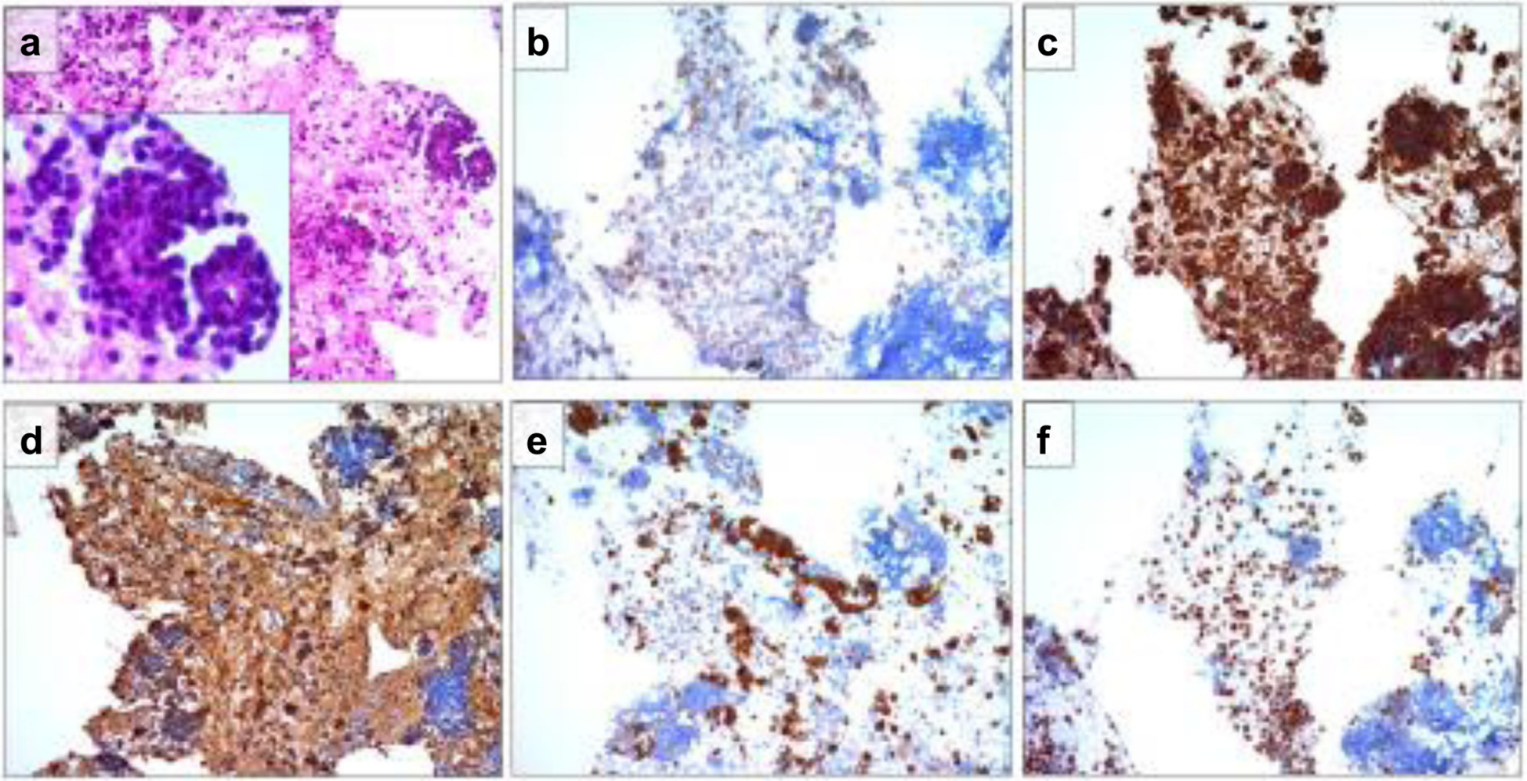
Endoscopic ultrasound-guided fine needle aspiration. **a** Cytological analyses revealed that the two masses were highly cellular and had papillary groups of small and uniform cells with oval nuclei surrounding a fibrovascular core. Immunohistochemistry was positive for **b** CD10, **c** α-1 antitrypsin, **d** vimentin, **e** neuron-specific enolase, and **f** progesterone receptor

Endoscopic ultrasonography (EUS) showed two indistinct and heterogeneous echo-poor masses (Fig. 2). The patient subsequently underwent EUS-FNA for each mass. On cytological analyses, the tumors were found to be highly cellular masses with papillary groups of small and uniform cells with oval nuclei surrounding a fibrovascular core. Immunohistochemically, the two masses were positive for α-1 antitrypsin, vimentin, neuron-specific enolase (NSE), CD10, and progesterone receptor. There was no histologic difference between the tumors. These features confirmed the preoperative diagnosis of multicentric SPNs (Fig. 3).

**Fig. 4:**
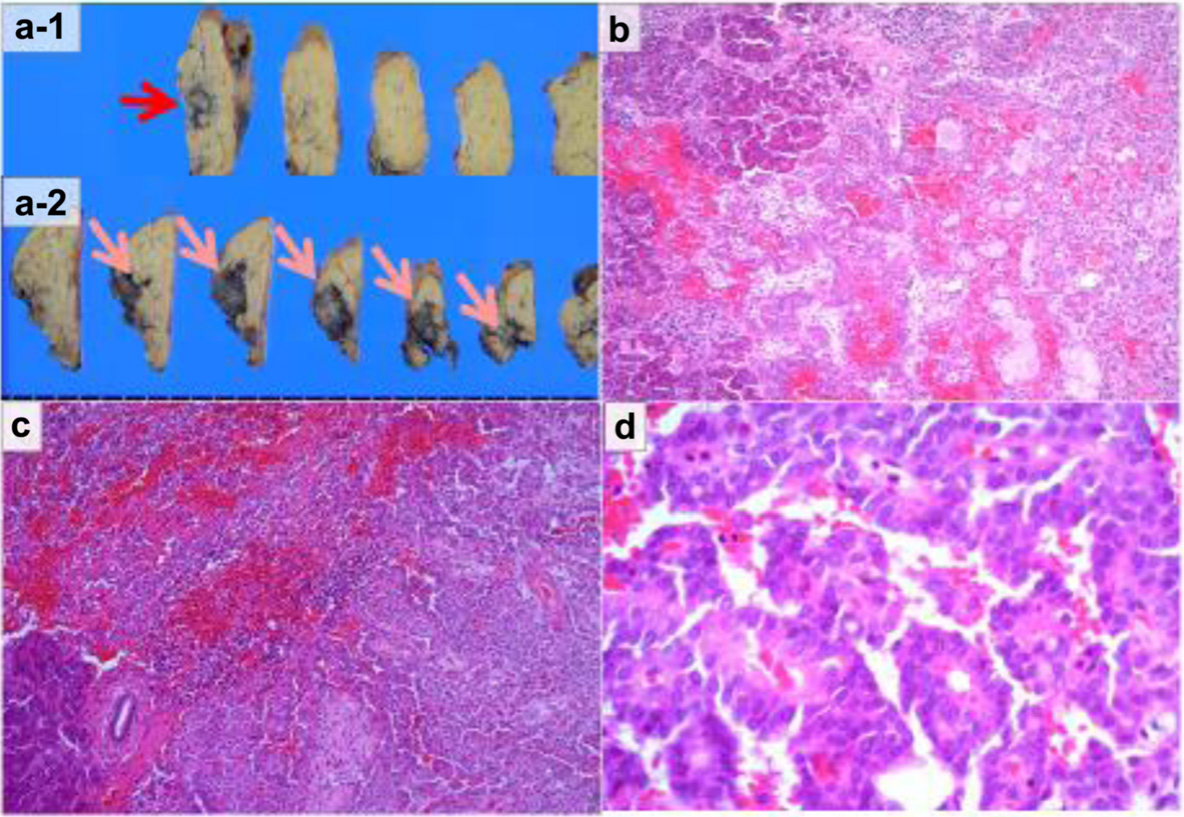
Histopathological findings. On macroscopic examination, both tumors were uncoated and solid lesions. (*a-1*) The tumor in the pancreatic body and (*a-2*) the tumor in the pancreatic tail. **b** Cytologic analysis revealed characteristic branching papillae with myxoid stroma in the tumor of pancreatic tail (hematoxylin and eosin [HE], ×100). **c** The tumor of the pancreatic body had the same characteristic features as the other tumor in HE staining (HE, ×100). **d** The tumor composed of small and uniform cells with oval nuclei surrounding a fibrovascular core (HE, ×400)

The patient underwent laparoscopic distal pancreatectomy (LDP). In order to achieve bleeding control, it became necessary to perform splenectomy because bleeding from the splenic vein developed while separating this vein from the pancreas. The final pathologic diagnosis was multicentric SPNs (Fig. 4). She recovered well during the immediate postoperative period and was discharged from the hospital 15 days later. During 2 years of follow-up, she has not developed any recurrence.

SPN is classified as an epithelial neoplasm of uncertain differentiation in the General Rules for the Study of Pancreatic Cancer, 6th Edition. Historically, SPN accounts for only 0.13–2.7 % of all pancreatic tumors and 1–2 % of exocrine pancreatic tumors [1]. SPNs usually occur in young women and have low malignant potential. In rare cases, SPNs may spread to the lymph nodes, resulting in distant metastasis. Therefore, complete surgical resection is necessary in patients with SPNs.

In this report, we have described a case of multicentric pancreatic tumors. The patient received a confirmed preoperative diagnosis of SPNs based on EUS-FNA findings, and she was therefore able to undergo laparoscopic surgery. SPN is usually a solitary mass, and multicentricity is exceptionally rare. To examine the rarity of multicentric SPNs, we performed searches of the PubMed database using the following keywords: (multicentric OR multi-centric OR two synchronous) AND (SPN). These searches revealed only 10 cases of multicentric SPNs that were reported recently (Table 1) [2–9]. Nine of these 10 cases involved two pancreatic tumors, as assessed based on abdominal CT scans; however, these patients did not receive a preoperative SPN diagnosis. Only one case [5] included EUS-FNA and a special stain of beta-catenin that suggested multicentric SPN. The present case is the first reported instance in which the patient received laparoscopic surgery.

**Table 1:** Recently reported cases of multicentric SPNs

	Case 1 [2]	Case 2 [3]	Case 3 [4]	Case 4 [5]	Case 5 [6]	Case 6 [7]	Case 7 [7]	Case 8 [7]	Case 9 [8]	Case 10 [9]	This case
Sex	Female	Female	Female	Female	Female	Female	Male	Female	Female	Female	Female
Age	26	17	31	34	24	57	56	44	24	16	32
Site	Head	Body	Head	Body	Head	Head	Body	Head	Head	Head	Body
	Tail	Tail	Tail	Tail	Tail	Body	Tail	Body	Tail	Tail	Tail
Size (cm)	2.3	3	8	3.7	5	1.5	3	4	Unclear	10	0.5
	9	6	19	2	8	3	12	11		7	1
Chief complaint	Abdominal pain	Abdominal pain	Abdominal pain	No	No	No	Abdominal mass	Abdominal mass	No	Abdominal pain	No
Preoperative diagnosis	Single mass	Not definite	Endocrine neoplasms	SPN	SPN considered	Not definite	Not definite	Endocrine carcinoma	SPN considered	Not definite	SPN
Treatment	TP	Unclear	DP	DP	DP	Unclear	DP	PD	LP	Tumor enucleation	LDP
			PD		PPPD				PD		

*TP* total pancreatectomy, *DP* distal pancreatectomy, *PD* pancreaticoduodenectomy, *PPPD* pulorus-preserving pancreaticoduodenectomy, *LP* left pancreatectomy, *LDP* laparoscopic distal pancreatectomy, *SPN* solid pseudopapillary neoplasm

In this case, it was difficult to diagnose SPN before surgery because the tumors had atypical features that did not resemble SPN: they were uncoated, solid, and multicentric lesions. CT scans are useful for diagnosing SPN, which is usually described as an encapsulated lesion with cystic degeneration on CT. However, small SPNs without cysts and capsules have been reported [1]. The present case involved two uncircumscribed tumors that were 5 and 10 mm in diameter, which may have been a reason for the atypical tumor features on CT. The differential diagnosis of multicentric pancreatic tumors can include endocrine neoplasms, intraductal papillary mucinous tumors, serous cystic neoplasms, mucinous cystic neoplasms, tumor-forming pancreatitis, pancreatic carcinoma, other complicated tumors, and metastatic pancreatic tumors. SPNs were considered in the differential diagnosis in this case because the patient was a young woman. However, a definitive diagnosis was not possible because the tumors were uncoated, solid, and multicentric lesions.

EUS-FNA is a useful diagnostic tool for pancreatic tumors—it has been reported to have a 91 % sensitivity and a 94 % specificity for diagnosing these tumors [10]. Song et al. found that the cytological features of SPN, as assessed by EUS-FNA, are highly characteristic and distinct from those of other cystic or solid pancreatic tumors [11]. Additionally, Law et al. reported that the addition of EUS-FNA increased the diagnostic yield to 82.4 %, as compared with CT (23.5 %), EUS (41.2 %), or CT and EUS (52.9 %) [12]. Furthermore, the overall complication rate of EUS-FNA was reported to be <1 % in large centers [13]. In one study, the complications of EUS-FNA included hemorrhage (0.96 %), acute pancreatitis (0.19 %), and duodenal perforation (0.09 %) [14]. It has generally been reported that EUS-FNA is a useful and safe method.

In recent years, the number of laparoscopic pancreatic procedures has increased because of the growing experience with laparoscopic surgery and availability of the relevant technology. LDP has gained worldwide acceptance because it does not require anastomosis or other reconstruction. In a recent comparison with conventional open surgery, LDP decreased blood loss and morbidity and promoted early recovery and shorter hospital stays [15]. It is important to confirm the LDP diagnosis before the operation; LDP should not be carried out in patients with invasive pancreatic carcinoma because the oncological consequences of laparoscopic pancreatic surgery remain quite controversial.

## Conclusions

We have reported a case of multicentric pancreatic SPN that was diagnosed preoperatively by EUS-FNA. No consensus exists on the prognosis and biological features of SPN. Thus, for tumors with atypical features that do not suggest SPN, EUS-FNA is a useful and safe method of definitively diagnosing SPN preoperatively.

## Consent

Witten informed consent was obtained from the patient for publication of this case report and any accompanying images.

## Abbreviations

CT: computed tomography
EUS: endoscopic ultrasonography
EUS-FNA: endoscopic ultrasound-guided fine needle aspiration
LDP: laparoscopic distal pancreatectomy
MRI: magnetic resonance imaging
NSE: neuron-specific enolase
SPN: solid pseudopapillary neoplasm
